# The Role of Complement Activation in Diabetic Nephropathy: Current Insights and Future Directions

**DOI:** 10.3390/jcm14238589

**Published:** 2025-12-04

**Authors:** Nikolaos Kotsalas, Ariadni Fouza, Maria Daoudaki

**Affiliations:** 1 Athens Naval and Veterans Hospital, Deinokratous 70, 11521 Athens, Greece; n.kotsal@yahoo.gr; 2 5th Surgical Department, Hippokration General Hospital, School of Medicine, Aristotle University of Thessaloniki, 54642 Thessaloniki, Greece; ariadnefou@gmail.com; 3 Laboratory of Biological Chemistry, School of Medicine, Faculty of Health Sciences, Aristotle University of Thessaloniki, 54124 Thessaloniki, Greece

**Keywords:** diabetic nephropathy, complement activation, urinary biomarkers, renal fibrosis, therapeutic targets, translational nephrology

## Abstract

Diabetic nephropathy (DN) is a leading cause of end-stage renal disease (ESRD) globally. Beyond metabolic and haemodynamic stress, the complement system has emerged as a contributor to glomerular and tubulointerstitial injury. In type 1 diabetes mellitus (T1DM), complement proteins contribute through autoimmune mechanisms, while in type 2 diabetes mellitus (T2DM) they are linked to insulin resistance. In both, complement activation promotes micro- and macrovascular complications through inflammatory pathways that accelerate DN progression. This review summarises the current evidence on the role of complement activation in diabetic nephropathy (DN). First, we outline the mechanisms by which the complement system is activated through the lectin pathway (in which mannoses bind to modified glycosylation structures), the classical pathway (in which C1q recognises immune complexes/damaged self), and the alternative pathway (in which C3 ticks over and amplifies on damaged renal surfaces). Next, we consider the roles of their effector molecules (C3a, C5a, and C5b-9/MAC), and the consequences of regulatory dysfunction (e.g., CD59 dysfunction). When integrated with findings from renal histology, blood and urine biomarkers enable us to evaluate the correlation between prognosis, disease severity, and progression. We will also discuss therapeutic implications, including the rationale behind selective complement inhibition and future intervention strategies.

## 1. Introduction

Diabetic nephropathy (DN) accounts for nearly 40% of renal failure cases in people with diabetes mellitus. Despite substantial therapeutic advances, a high proportion of patients remain at risk for renal disease progression, suggesting the role of non-metabolic and immunological mechanisms in the pathogenesis of DN [1,2,3,4].

DN was formerly considered to be a metabolic and haemodynamic complication of diabetes [4,5]. However, recent studies have re-evaluated this perspective, with the current consensus being that it is an immunometabolic disorder characterised by glomerular and tubulointerstitial injury [6,7]. A distinctive correlation between hyperglycaemia and immune-related inflammation is recognised, distinguishing this condition from other chronic kidney diseases (CKDs) [8]. Hyperglycaemia is considered a significant contributing factor to renal injury, through multiple interconnected mechanisms, including oxidative stress [9], the polyol and hexosamine metabolic pathways [10], protein kinase C (PKC) activation, the TGF-β signalling pathway [11], and the accumulation of advanced glycation end products (AGEs) [12,13]. Further amplifying factors are epigenetic modifications, impaired autophagy, mitochondrial dysfunction and the release of pro-inflammatory cytokines, including TNF-α, IL1, and IL 6 [12].

AGEs result from a non-enzymatic reaction of proteins with sugars and have been observed to accumulate and modify the basement membrane of the glomeruli, mesangial cells, endothelial cells, and podocytes in patients with diabetes and/or end-stage renal disease [14]. It has been demonstrated that the aforementioned modifications generate “altered-self” epitopes, as a result of the complement system activation, primarily via the lectin pathway and, in a secondary manner, via the classical pathway [15,16].

Focusing on investigations specific to diabetic nephropathy, experimental and clinical data have demonstrated that intrarenal complement activation is a hallmark feature of biopsy-proven diabetic nephropathy [17,18]. Proteomic, transcriptomic, and immunohistochemical analyses have revealed the presence of C3 deposition and its fragments within the glomeruli and tubules [19,20,21]. These data also demonstrated elevated levels of C3 peptides in both plasma and urine. The correlation of these complement products with albuminuria, histopathological injury, and renal decline provided robust evidence to substantiate immune-mediated damage. Consequently, complement dysregulation in DN represents a pivotal pathogenic mechanism, which contributes to progressive fibrosis and renal failure [20,22].

A literature search was conducted in PubMed, Scopus, and Web of Science to ensure a comprehensive overview of complement activation in diabetic nephropathy. The search covered publications from January 2000 to October 2024 and used the Boolean string: “diabetic nephropathy” AND (“complement” OR “complement system” OR “complement activation” OR “C3a” OR “C5a” OR “C5b-9” OR “membrane attack complex”). Additional relevant articles were identified through the reference lists of selected studies. Because some recent studies (2023–2025) use the broader term diabetic kidney disease (DKD), and DN represents a more specific clinical and histopathological entity, we restricted our search to DN for reasons of terminological and scientific precision.

## 2. DN Overview

Globally, diabetes mellitus (DM) has surpassed hypertension and glomerulonephritis as the primary cause of CKD [23]. Among patients suffering from ESRD, DN accounts for approximately 28% of cases, compared to 25% for hypertension and 22% for glomerulonephritis [6]. It develops in about 15–25% of patients suffering from type 1 diabetes mellitus (T1DM) and in 30–40% of patients suffering from type 2 diabetes mellitus (T2DM) [24,25,26,27].

The classic Mogensen model (1983) [27] delineates five progressive stages of DN:

1. Renal hyperfunction and hypertrophy;
2. Mesangial expansion;
3. Onset of microalbuminuria;
4. Overt proteinuria, which may or may not be accompanied by nodular glomerulosclerosis;
5. End-stage fibrosis.

However, atypical trajectories have been observed, with some patients experiencing a progressive decline in estimated glomerular filtration rate (eGFR) preceding microalbuminuria [28,29,30], despite the therapeutic progress that has been achieved [31].

The role of the complement system in the pathogenesis of DN is well-documented, supported by the presence of complement components in serum, urine, and renal biopsies of diabetic patients, correlating with disease severity [18,32,33]. Without effective treatment, DN may progress to ESRD within approximately seven years, although only 30–45% of patients demonstrate progression over a period of a decade, while others remain stable [25]. The American Diabetes Association (ADA) recommends annual DN screening: from five years after T1DM diagnosis, and at diagnosis for T2DM [34,35]. As discussed earlier, management focuses on strict glycaemic and blood pressure control, lipid regulation, and selective protein restriction. Nevertheless, the prevalence of ESRD has nearly doubled in the United States over the past decade [34], with a similar trend seen in Europe [36], thereby emphasising the necessity for preventive strategies and novel therapeutic targets.

## 3. Complement System Overview

The complement system comprises over 30 plasma and membrane proteins [37,38,39]. Following activation by the three pathways (alternative, classical, and lectin), these proteins interact to detect and interact with altered or foreign surfaces, resulting in opsonisation, inflammation, and cell lysis [40]. The C1q protein component of the classical pathway binds to immune complexes or altered autoantigens, and cleaves factors C4 and C2, forming the enzyme C3 classical convertase (C4b2a) [41]. This enzyme is also formed in the lectin pathway, which is triggered by particular carbohydrate or glycosylation patterns. This activates MBL-associated serine proteases (MASP-1 and MASP-2), which leads to C4b2a convertase [42,43,44].

The alternative pathway involves continuous low-level “function” of C3. C3b is produced, which then binds to factors B and D to form the C3bBb convertase. This enhances complement activation on unprotected or damaged surfaces [45].

**Figure 1:**
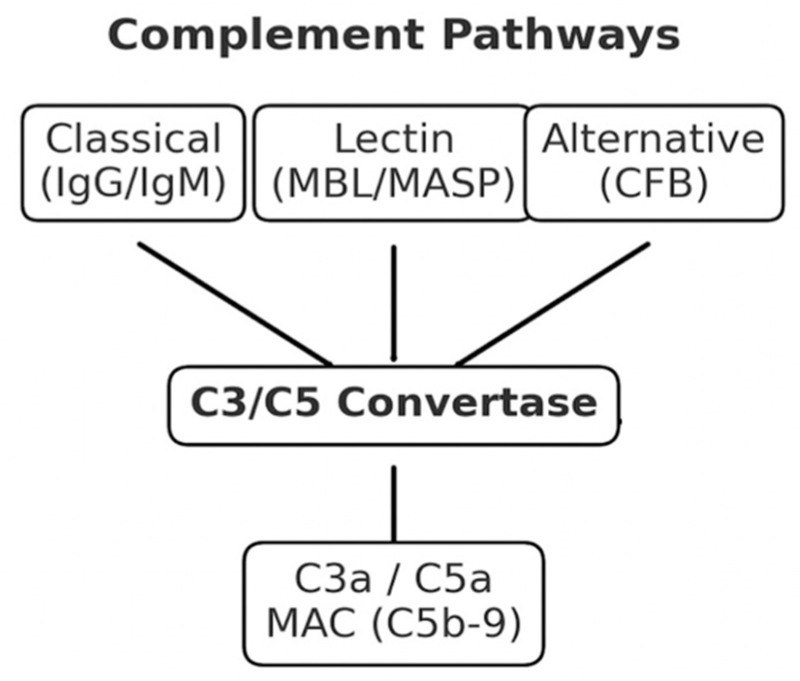
Schematic representation of pathways of complement activation. It shows the classical, lectin and alternative pathways, how they converge at C3 and C5, and how effector molecules (C3a, C5a and C5b-9) are generated.

Classical and alternative convertases cleave C3, as shown in Figure 1, and C3a and C3b are produced, followed by C5a and C5b. The latter contributes to the assembly of the membrane attack complex (C5b-9, or MAC), which can cause lytic or subclinical damage [46]. Complement activation is strictly controlled by regulatory factors, including CD46, CD55, CD59, factor H, and factor I, which prevent excessive activation and protect the host [41,47].

## 4. Pathway-Specific Activation in DN

Evidence for the activation of the complement in DN indicates that the lectin pathway has been identified as the primary initiator of complement activation, with the alternative pathway also being activated and the classical pathway contributing to specific pathological contexts [48,49,50,51].

## 5. The Lectin Pathway

Alterations in protein glycosylation specific to diabetes, particularly non-enzymatic glycation and modified N-glycan branching in diabetic tissues, result in the formation of advanced glycation end products (AGEs). These AGEs alter the molecular patterns of self in renal structures that are easily recognised by pattern recognition molecules. These include lectins such as mannose-binding lectin (MBL) and ficolins [52,53,54,55], which activate the lectin complement pathway and result in the formation of C4b2a convertase [56,57]. Therefore, these glycosylation abnormalities are a key driver of lectin pathway activation in diabetic nephropathy. Consequently, lectins serve as vital pattern recognition molecules that link hyperglycaemia-induced biochemical alterations to innate immune activation. This places the lectin pathway at the centre of complement dysfunction in DN.

Clinical and experimental studies have indicated that serum MBL concentrations are higher in cases of DN than in diabetes without nephropathy [52,58,59] and are correlated with albuminuria and a decline in renal function [60]. Genetic studies have established a correlation between specific *MBL2* gene variants, which are associated with elevated MBL serum levels, and an augmented risk of DN progressing to ESRD [61,62,63]. Immunohistochemical data from preclinical models also confirm local lectin pathway activation in biopsy-proven DN patients. These studies show MBL deposition in the glomeruli [64,65,66,67], but direct MASP deposition in patients has not yet been confirmed.

Taken together, these findings demonstrate that the lectin pathway activation is not merely a secondary response to tissue injury in DN, but rather a primary immunometabolic mechanism that senses and amplifies the biochemical alterations induced by diabetes.

## 6. Classical Pathway

The classical pathway is responsible for a secondary, albeit measurable, function in DN. Although immune complexes is not a hallmark of diabetic nephropathy, C1q and C4d deposits have been identified in renal biopsies from patients with DN. These deposits have been shown to correlate with a faster decline in renal function and a poorer prognosis [18,19]. The presence of both C1q and MBL in certain specimens indicates the potential for cross-activation between the classical and lectin pathways [56]. Consequently, the classical pathway may be particularly significant in advanced or inflammatory stages of DN, amplifying complement deposition initiated by other triggers.

## 7. Alternative Pathway

The primary function of the alternative pathway is to act as a feedback loop, thereby ensuring the maintenance of complement activation initiated by the lectin or classical pathways. Low-level spontaneous hydrolysis of C3 produces C3bBb convertase, which continuously cleaves C3 on unprotected surfaces [45,68]. Glycosylated basement membranes and oxidatively modified tubular cells are ideal substrates for this amplification process [14,32]. Renal transcriptomic analyses and immunostaining have revealed the upregulation of factor B (CFB) and properdin in DN, particularly within the glomeruli and proximal tubules [69]. High glucose levels induce CFB via mTORC1/STAT1 pathway signalling, highlighting its important role in DN [69]. Glycation and oxidative stress disrupt the regulatory mechanisms catalysed by CD46, CD55, CD59, factor H, and factor I, leading to sustained complement activation within the glomeruli and tubulointerstitial space. This results in podocyte apoptosis and endothelial dysfunction with accelerating renal fibrosis [70].

Assuming that urine reflects intrarenal activity, including immune activity, capillary electrophoresis-mass spectrometry (CE-MS) and liquid chromatography with tandem mass spectrometry (LC–MS/MS) can accurately detect complement-derived peptides in urine. Results of those techniques established a reproducible proteomic signature of DN progression, with C3-, C4- and C5-derived peptide enrichment, particularly C3a, C3dg, C5a, and sC5b-9, in DN patients compared with diabetic controls without nephropathy [71,72,73,74], as detailed further in the urinary proteomics section. Clinically, the levels of these fragments correlate with albuminuria, histological severity, and diminished eGFR, indicating that urinary complement components predominantly reflect intrarenal activation rather than a systemic effect [18,32]. Additionally, targeted assays also identified C7, factor B and properdin peptides in the urine of DN patients, which is consistent with active participation of the alternative and terminal phase of complement activation within the kidney [75]. In line with the amplification role of the alternative pathway, urinary concentrations of C3a and C5a, as well as soluble C5b-9 (sC5b-9), increase progressively from the early to advanced stages of DN [76]. These increases may serve as non-invasive indicators of ongoing intrarenal complement activation [77,78] though the role of sC5b-9 remains uncertain [79].

Collectively, these results suggest that the alternative pathway acts as a potent amplifier of intrarenal complement activation, converting it into chronic inflammation and fibrosis in GN.

## 8. The Role of Effector and Regulatory Molecules in the Activation of the Complement System in DN

Following complement activation, the anaphylatoxins C3a and C5a are produced, along with the membrane attack complex (MAC) C5b-9 [46,80].

## 9. C3a and C5a Are Pro-Inflammatory and Profibrotic Anaphylatoxins

The pro-inflammatory anaphylatoxins C3a and C5a, generated by C3 and C5 cleavage bind to their respective receptors C3aR and C5aR1, on podocytes, mesangial cells, tubular epithelial cells, and infiltrating macrophages [76,80,81,82]. The binding of C3a and C5a to their receptors, activates the NF-κB, MAPK, and PI3K/Akt signalling pathways, which in turn drive oxidative stress, mitochondrial dysfunction, cytokine release, and profibrotic structural changes [83,84,85,86].

Experimental models have provided evidence that C5a–C5aR1 binding gives signals to augment tubular injury by upregulating the NF-κB-dependent expression of MCP-1, IL-6, and TGF-β, thereby promoting the recruitment of macrophages and the accumulation of proteins of the extracellular matrix [76,87]. Conversely, pharmacological blockade or genetic deletion of C5aR1 has been shown to mitigate glomerular inflammation, lower albuminuria, and preserve renal architecture [88]. C3a-C3aR binding give signals to accelerate epithelial–mesenchymal transition and collagen synthesis, thereby contributing to tubulointerstitial fibrosis [89].

## 10. C5b-9 (Membrane Attack Complex)

In the context of diabetic nephropathy, C5b-9 deposition occurs in both the glomerular and tubular compartments, often aligning with mesangial expansion, tubular atrophy, and interstitial fibrosis [66,90]. While heavy MAC deposition can cause direct cytolysis, sublytic C5b-9, which predominates in chronic DN is pathologically salient [91], triggers an influx of calcium ions within the cell, in addition to the activation of PI3K/Akt pathways, thereby enhancing the TGF-β production and downstream profibrotic pathways [92,93]. Fibroblasts are activated followed by stimulation of extracellular matrix protein deposition, driving glomerulosclerosis and tubulointerstitial fibrosis, independently of overt cell death [94,95,96]. In a clinical context, the intensity of C5b-9 staining in renal biopsies has been shown to correlate with the disease stage and a reduction in renal function, supporting its role as a tissue biomarker of complement-induced damage [90,97]. Therefore, MAC not only functions as the endpoint of complement activation, but also as an active mediator that links sustained complement activation to chronic inflammation and fibrogenesis in DN.

## 11. C7 and Additional Regulatory Proteins

The complement component C7, a MAC component, is upregulated in early-stage diabetic nephropathy in both serum and kidney tissue compared to non-diabetic controls [98].

Notably, the localisation of C7 in the mesangium and its regulation by microRNAs (miRNAs) miR-494-3p and miR-574-5p provided evidence for an intrinsic renal source of complement synthesis [99]. This local C7 synthesis can facilitate MAC assembly within glomerular structures even before extensive systemic activation.

In the context of diabetes, the regulatory mechanisms that usually limit complement activation are often impaired. Beyond CD59 glycation, which impairs MAC inhibition, analyses of human DN kidneys revealed reduced expression of the regulatory proteins CD46 (MCP) and CD55 (DAF) [100,101,102]. This leads to uncontrolled C3/C5 convertase activity. The collective loss of these checkpoints enables persistent intrarenal activation and inflammation, thereby reinforcing the transition from metabolic stress to chronic tissue injury.

Together, C7 upregulation and loss of CD46/CD55/CD59 function generate an environment of uncontrolled complement activity, exacerbating injury and fibrosis beyond classical MAC deposition [103].

In summary, the complement activation phase in DN integrates the inflammatory components C3a and C5a, the cytotoxic/sublytic component C5b-9, and the regulatory components CD59, CD46, and CD55 into a unified pathogenic axis. Anaphylatoxins lead to the recruitment of immune cells and the promotion of fibrogenic signalling, while subclinical lytic MAC assembly promotes structural reprogramming under defective regulatory control. Table 1 shows the involvement of all complement components in DN.

Excessive activation, combined with impaired regulation and signal transduction failure, is the cause of complement-mediated damage in DN, highlighting the therapeutic potential of interventions that selectively target the complement system.

Histological Correlates: complements fragment deposition and morphological change.

Complement components C1q, C3, C4d, and C5b-9 (MAC) are frequently co-localised within glomerular and tubular compartments in renal biopsies from DN patients [18,63]. In patients with classical or lectin pathway activation, C4d staining is often observed along the glomerular capillary walls, alongside mesangial expansion and nodular sclerosis and in Bowman’s capsule [18,104,105]. C3 deposition in the glomerulus is common, predominantly mesangial, and its presence increases with higher Renal Pathology Society classes and is associated with worse renal outcomes [106]. C5b-9 preferentially accumulates in the mesangium and capillary wall, with staining intensity paralleling histological severity, interstitial fibrosis, tubular atrophy, and accelerated decline in renal function [90]. In cases exhibiting inflammatory infiltrates, C3aR^+^ and C5aR1^+^ macrophages are often detected, which is consistent with local chemoattractant signalling and injury propagation [87,107,108].

## 12. Urinary Proteomics: Non-Invasive Fingerprint of Intrarenal Complement Activation

As discussed in the Alternative Pathway section, sC5b-9 serves as a composite marker linking complement activation to tubular injury and epithelial stress [86].

## 13. Serum Proteomics: Complement Dysregulation and Disease Monitoring

Although intrarenal complement activation dominates the pathophysiology in DN, systemic alterations in complement component levels are also measurable. Serum proteomics and ELISAs have reported increased levels of C3a, C5a, and C7, as well as decreased levels of factor H, indicating insufficient regulatory control in the circulation [109], causing predisposition to spontaneous activation and persistent low-grade inflammation. Consistent with findings described in the C7 section, serum and renal C7 upregulation supports its role as a dual-compartment biomarker for early detection and disease trajectory assessment [98,99]. Elevated levels of serum MBL have been associated with the onset and progression of microalbuminuria. This is indicative of systemic lectin pathway activation, which in turn contributes to renal function impairment. From a clinical perspective, integrating serum and urinary complement profiles can help distinguish systemic from intrarenal complement activity, as discussed in the Integrated Interpretation section.

## 14. Biopsy and Tissue Proteomics: Local Complement Synthesis in the Kidney

Studies using transcriptomics and single-cell RNA sequencing establish that the kidney is not merely a target of complement activation, but also a source of complements. Mesangial cells, podocytes, and proximal tubular epithelial cells from DN patients express C3, C7, factor B, and properdin mRNA, demonstrating local complement synthesis and in situ amplification capacity [99,110]. Laser-capture microdissection coupled to proteomics, has shown that sclerotic and fibrotic regions are enriched in complement components, particularly C3, C7, and other MAC subcomponents [111]. This aligns with the upregulation of TGF-β and collagen IV deposition [112]. Multi-omics maps further reveal that complement gene networks cluster with genes responsible for oxidative stress, cytokine signalling, and extracellular matrix (ECM) remodelling programmes, confirming the close coupling of immune, metabolic, and fibrotic pathways in DN [113].

## 15. Integrated Interpretation and Clinical Implications

Complement activation is a robust indicator of structural damage and functional renal deterioration in DN, as evidenced by histology, urinary/serum proteomics, and transcriptomics.

Histologically, complement deposition parallels glomerulosclerosis and interstitial fibrosis, while urinary proteomics reflects real-time intrarenal activation, identifying early biomarkers, such as C3a, C5a, and sC5b-9.

Serum assays reveal systemic dysregulation and pathway bias, notably in the lectin and alternative pathways, and tissue omics confirm that renal cells actively synthesise and amplify complement components.

Together, diabetic metabolic injury, complement activation, and fibrotic remodelling create a self perpetuating immune metabolic feedback loop, sustaining inflammation despite optimal glycemic and haemodynamic control and highlighting new opportunities for diagnosis and therapy. Emerging technologies such as spatial transcriptomics, advanced mass spectrometry-based proteomics, and urinary multimarker panels will further refine patient risk stratification and enable biomarker-guided complement therapies in cases of diabetic nephropathy (DN).

**Implications for clinical practice and future aspects:**

The management of DN is characterised by glycaemic control, optimisation of blood pressure, and the inhibition of the renin–angiotensin–aldosterone system (RAAS). The incorporation of SGLT2i inhibitors in conjunction with MRAs (non-steroidal mineral corticoid receptor antagonists) has further improved renal and cardiovascular outcomes. Despite these advances, residual renal risk remains a problematic issue even in well controlled patients. This can be attributed to the heterogeneity among DN patients, characterised by different combinations of albuminuria, glomerular, and tubular damage, as well as activation of innate immunity, with complement activation being important. In view of the fact that current therapeutic management practices have not adequately addressed the immune and inflammatory pathways responsible for disease progression in certain phenotypes, it is imperative to recognise this heterogeneity to facilitate the integration of complement activation with clinical phenotypes and therapeutic pathways.

From a clinical perspective, complement-related biomarkers have the potential to improve risk stratification in DN patients. Combining urinary complement biomarkers including C3a, C5a, and soluble C5b-9 (sC5b-9) with established kidney disease markers such as albuminuria, eGFR slope, and tubural injury proteins (e.g., NGAL) may help to identify patients with intrarenal complement activation who are at a higher risk for disease progression. In certain cases, complement deposition patterns in kidney biopsies (e.g., C4d, C5b-9) can support the identification of complement-driven disease subtypes in conjunction with histopathological evaluation.

Given the success of complement inhibitors in treating other renal diseases, they could provide a potential framework for the future therapeutic modulation in DN. For example, C5a-mediated signalling could be inhibited by blocking its binding to C5aR1 with avacopan, as has been successfully shown in ANCA vasculitis. Furthermore, inhibitors of the terminal pathway such as eculizumab and ravulizumab could be used. If there is a predominance of alternative pathway activation, an inhibitor of factor B, such as iptacopan, could also be considered, as has been performed in IgA nephropathy. Alternatively, a general inhibitor of the C3 complement component such as Pegcetacoplan, could be used. 

Patients demonstrating lectin or alternative pathway activation, marked urinary complement peptide excretion, or extensive complement deposition may be suitable candidates for future clinical trials. In these trials, complement biomarkers could be used to select patients and monitor complement pathway inhibition.

Future research should focus on validating complement-based biomarker assays in their large prospective cohorts and integrating them into existing risk models. Standardising urinary and serum assays, determining clinically actionable thresholds and assessing results across diverse populations will be crucial for clinical translation.

Ultimately, well designed trials are needed to test whether adding complement-targeted therapies to the current standard of care can slow eGFR decline or prevent adverse renal outcomes in individuals with complement-driven DN. If such strategies are proven to be effective, they would support a precision medicine approach in which the complement functions as both a mechanistic driver and a therapeutic target.

## 16. Metabolic–Complement Crosstalk in Diabetic Nephropathy

Metabolic dysregulation and complement activation are tightly interconnected processes in DN. Persistent hyperglycaemia leads to the formation of AGEs and reactive oxygen species, which modify renal basement membranes and activate the lectin and alternative complement pathways. Conversely, complement effectors such as C3a, C5a, and sublytic C5b-9 amplify cellular stress by enhancing transforming growth factor-β (TGF-β) and connective tissue growth factor (CTGF) signalling in tubular and glomerular cells, contributing to chronic kidney disease (CKD) progression.

## 17. Conclusions

Diabetic nephropathy is now recognised as a chronic immunometabolic disease in which metabolic and immunological pathways converge to drive progressive renal damage. Complement activation acts as a key amplifier of glomerular and tubular damage in this condition, linking hyperglycaemia to inflammatory and fibrotic responses. Multi-omics and histopathology analysis demonstrate a strong correlation between complement dysregulation, disease activity, and renal functional decline.

The lectin pathway dominates intrarenal activation, with the classical pathway contributing less significantly to inflammatory progression. Key molecules involved in this process include C3a, C5a, C7, and C5b-9, and these molecules mediate cellular damage and profibrotic responses, while glycation of complement regulators CD59, CD46, and factor H eliminates critical control points, thereby enabling chronic, low-grade complement activation.

Urinary complement components, such as C3a, C5a, C7, and sC5b-9, when combined with traditional markers such as albuminuria, eGFR slope, and tubular stress proteins (KIM-1, NGAL), may enable complement-based risk stratification and identification of patients with complement-driven disease activity.

Complement biomarkers could contribute to the development of precision therapies by supporting the development of targeted complement inhibitors. The incorporation of such biomarkers into observational and interventional studies could lead to the stratification of DN patients based on these markers. Their standardisation and validation in prospective cohorts will be essential to translating complement biology into clinical benefits.

## Figures and Tables

**Table 1:** Complement components implicated in diabetic nephropathy:

Complement Component	Pathway/Function	Role in DN
C1q/C4/C2	Classical pathway initiation	Activated by glycated or oxidised self-antigens; promotes glomerular inflammation.
MBL/MASP-1/MASP-2	Lectin pathway	High levels in diabetic serum and kidneys; correlation with albuminuria and DN progression.
C3	Central activation molecule (all pathways)	Overexpressed in glomeruli and tubules; C3a drives inflammation and fibrosis; urinary C3 fragments predict eGFR decline.
C5/C5a/C5b-9 (MAC)	Terminal phase	Promotes mesangial and tubular injury; sublytic MAC induces cytokine and extracellular matrix production.
C7	MAC component	Anchors MAC to cell membranes; overexpressed in DN, linked to glomerular and interstitial damage.
Factor B/Properdin	Alternative pathway amplification	Upregulated in diabetic kidneys; enhances C3 convertase stability and local complement deposition.
Factor H	Soluble regulator of the alternative pathway	Glycation impairs function, leading to uncontrolled complement activation; reduced serum and urinary levels in DN.
CD55 (DAF)	Membrane-bound inhibitor of C3/C5 convertases	Downregulated under hyperglycemia; its loss of expression increases complement-mediated cytotoxicity.
CD59	Membrane-bound inhibitor of MAC formation	Reduced expression in diabetic kidneys; loss of expression correlates with C5b-9 deposition and fibrosis severity.

## Data Availability

This article is a review and does not include original data.
